# Identification and characterization of the *KNOTTED1-like* homeobox as an active regulator of leaf development in chickpea

**DOI:** 10.3389/fpls.2026.1803976

**Published:** 2026-03-17

**Authors:** Natalia Gutierrez, Patricia Castro, Josefa Rubio, Jose V. Die

**Affiliations:** 1 Área de Mejora Vegetal y Biotecnología, IFAPA-Centro Alameda del Obispo, Córdoba, Spain; 2 Departamento de Genética, Escuela Técnica Superior de Ingeniería Agronómica y de Montes (ETSIAM), Universidad de Córdoba, Córdoba, Spain

**Keywords:** class M, KASP marker, KNOX, leaf morphology, near-isogenic lines (NILs)

## Abstract

Leaf architecture is a key factor influencing plant productivity. In chickpea (*Cicer arietinum* L.), leaf type varies from the typical compound form to simple-leaf mutants. Previous studies have suggested a monogenic inheritance pattern for leaf type, although the causal genes remain unidentified and functionally uncharacterized. Despite advances in understanding leaf development in other legumes, the molecular basis of leaf morphology in chickpea remains poorly explored. Using an integrative strategy combining phenotyping, genetic analysis, and high-throughput sequencing, we developed near-isogenic lines (NILs) segregating for leaf type to refine the genomic region underlying this trait. Variant discovery revealed a strong candidate gene, LOC101500499, annotated as a *homeotic knotted-1-like* (*KNOX*) gene on chromosome 8. A single-nucleotide deletion within the open reading frame was identified exclusively in simple-leaf genotypes, generating a frameshift and premature stop codon. Transcriptomic comparisons between compound- and simple-leaf plants showed significant differences in *KNOX* expression levels consistent with the mutation’s predicted functional impact. To validate the association, we designed a KASP marker targeting the deletion, which accounted for 90% of phenotypic variation in leaf type across diverse materials. Conserved domain analysis and phylogenetic classification indicated that the encoded protein belongs to the M subclass of the *KNOX* gene family, lacking the ELK and homeodomain regions typical of classes I and II. The discovery of a causal deletion in the *knotted-1-like KNOX gene* linked to the simple-leaf phenotype, together with its robust validation through KASP genotyping, provides a valuable molecular tool for breeding and deepens our understanding of leaf morphology in the species. Further studies on the regulatory networks interacting with this gene will help elucidate the mechanisms governing compound leaf development in chickpea.

## Introduction

Efficient use of solar radiation is essential for optimal crop productivity. To achieve this, a significant portion of the light must be captured by photosynthetic tissues, primarily the leaves. As the main organs responsible for both light interception and photosynthesis, leaves play a crucial role in plant growth and biomass accumulation. Consequently, modifications in plant architecture, particularly changes in leaf shape, can influence the agronomic performance of a genotype. In angiosperms, leaves are classified as either simple or compound. Simple leaves consist of a single blade attached to a petiole, while compound leaves are composed of multiple blade units, known as leaflets, arranged along a central rachis. This variation in leaf architecture has drawn considerable interest in recent years, particularly regarding its molecular and genetic regulation.

Chickpea (*Cicer arietinum* L.), a self-pollinating annual legume in the Papilionoideae subfamily, ranks as the third most widely cultivated grain legume, with an annual production of 16.5 million tons over 14.1 million hectares, yielding an average of 1.17 t/ha in 2023 (FAOSTAT, 2023). The species has a genome size of approximately 738 Mb (2n = 2x = 16), with the reference genome of the CDC Frontier kabuli cultivar assembled into 530 Mb (Varshney et al., 2013). The cultivated chickpea typically develops compound leaves that are either imparipinnate or pseudo-imparipinnate, characterized by a terminal leaflet not occupying a true terminal position (Cubero, 1987). These leaves generally consist of 9 to 15 alternately arranged leaflets on a 3–7 cm long rachis. However, several leaf shape mutants (simple or unifoliate and multipinnate) have been identified that alter the size, shape, or arrangement of leaflets (Rao et al., 1980; Pundir et al., 1990; Singh and Weigand, 1996; Gaur and Gour, 2003; Danehloueipour et al., 2008; Khan et al., 2011). Agronomic studies have shown that compound-leaved chickpea varieties generally produce higher seed yields than unifoliate types. This is largely because plants with pinnate leaves tend to have a higher number of pods per plant, which directly translates to more seeds (Toker et al., 2012). Nevertheless, the unifoliate (simple leaf) mutant has gained particular attention in breeding programs due to its association with larger seed size. Thus, this trait has been successfully incorporated into commercial cultivars in several countries, including Surutato-77 and Macarena in Mexico; Sierra, Dwelley, Sanford, Evans, and UC Pegasus in the USA; CDC Diva and CDC Xena in Canada; and Kimberley Large in Australia (Warkentin, 2003; Siddique and Regan, 2005; Danehloueipour et al., 2008). To better understand the genetic basis of leaf type variation, genetic approaches have been utilized to map *loci* associated with this trait. Early studies on simple and compound leaves in chickpea suggest a monogenic mode of inheritance (Rao et al., 1980). Specifically, two genes, designated *ml* and *sl*, have been reported to regulate leaf type in chickpea. The dominant alleles of both genes result in the typical uni-imparipinnate leaf form, whereas the loss of dominance at the *ml* locus leads to the development of simple leaves (Pundir et al., 1990; Toker et al., 2012). These genes are yet to be physically mapped and functionally characterized for understanding the crosstalk between them. While significant advances have recently been made in specific aspects of leaf development, such as the sequential progression of leaflet formation (Liu et al., 2023), comprehensive studies addressing the molecular basis of leaf morphology in chickpea are still lacking. This gap in knowledge is particularly notable when contrasted with the considerable interest and progress made in understanding leaf morphological diversity in other legumes.

The *KNOX* (*KNOTTED-like HOMEOBOX*) gene family represents a highly conserved group of genes in plants that play a crucial role in regulating leaf development (Jia et al., 2023). The *KNOX* genes are grouped into three classes based on amino acid sequence identity, domain organization, and expression patterns: Class I, Class II, and Class M (Kerstetter et al., 1994; Magnani and Hake, 2008; Peng et al., 2011). KNOX proteins are known to contain four conserved structural domains, each with distinct biological functions: a C-terminal homeodomain (HD), KNOX1 and KNOX2 domains (together referred to as the MEINOX domain) in the conserved N-terminal region, and an ELK domain located upstream of the homeodomain. Class I and Class II KNOX proteins are present in all land plants, and KNOX homologs can even be found in green algae. In contrast, the class M proteins arose later in plant evolution and are restricted to eudicots (Gao et al., 2015). Among these three classes, Class I KNOX genes have been extensively studied, whereas the functions of Class II KNOX genes remain largely unresolved, and the M class has received virtually no attention.

In this study, we identified and characterized a candidate gene for leaf type using an integrative approach that combined phenotyping and genetic analyses, including the development and sequencing of plant material (recombinant inbred lines and a pair of near-isogenic lines), variant calling, gene expression analysis, conserved domain characterization, functional annotation and evolutionary relationship analysis. Based on these data and validation of the allelic diversity of this candidate, we propose the *homeobox protein knotted-1* (LOC101500499) as a strong candidate gene controlling compound leaf formation in *C. arietinum*.

## Materials and methods

### RIL population and NIL development

A total of seventy-seven F_6:7_ chickpea recombinant inbred lines (RILs), derived from an intraspecific cross between the genotypes CA2990 (female parent, simple leaf; SL) and WR315 (male parent, compound leaf; CL), were used in this study. This population, designated as RIP16 (Recombinant Inbred Population 16), was developed at IFAPA (Andalusian Institute of Agrarian and Fishing Research and Training, Spain), through the Single Seed Descent (SSD) method. The parental line CA2990 is a Mexican kabuli chickpea maintained at IFAPA, while WR315 is a desi landrace conserved at ICRISAT (International Crops Research Institute for the Semi-Arid Tropics, India). Among the lines in RIP16, RIL28 exhibited residual heterozygosity for leaf type and was selected for the development of a pair of near-isogenic lines (NILs). For this purpose, fifteen seeds from RIL28 were sown and evaluated. A heterozygous plant for leaf morphology was identified and selected in each generation. This selection process was repeated over multiple cycles, from 2018 to 2022, until progenies showed no segregation for leaf type. Finally, one homozygous descendant for each leaf type was self-pollinated once more to ensure genetic stability. These were considered the resulting NILs, sharing an almost identical genetic background except for the leaf morphology: NH16/28-CL (compound leaf) and NH16/28-SL (simple leaf).

### Phenotypic characterization

Phenotypic evaluation of the 77 individuals from the RIL population was conducted in multiple growing seasons (from 2018 to 2022) under field conditions at the IFAPA experimental station in Córdoba, Spain (latitude 37°53’N, longitude 4°47’W, altitude 117 m). The primary trait evaluated was leaf type, classified as either *simple* or *compound*. In addition to leaf morphology, leaf size was assessed by measuring the leaf surface area (mm²). For this, three green leaves from the fourth node were collected from three randomly selected plants per RIL. Digital images of the leaves were obtained using a flatbed scanner, and the surface area was quantified using SigmaScan Pro 5.0 imaging software.

### DNA extraction and resequencing

Total genomic DNA was extracted from individual young leaf tissue of the parental lines WR315 (compound leaf; CL) and CA2990 (simple leaf; SL), as well as from the two near-isogenic lines (NILs), NH16/28-CL and NH16/28-SL. The extraction was performed using the DNeasy Plant Pro Kit (Qiagen), following the manufacturer protocol. Whole-genome resequencing (WG-Seq) was carried out at the Centro Nacional de Análisis Genómico (CNAG-CRG) in Barcelona, Spain. Sequencing was performed on an Illumina NovaSeq 6000 platform, generating paired-end reads of 2 × 150 bp, with an average output of 16 Gb per sample. Raw sequence data were processed using Illumina Sequencing Analysis Viewer, and underwent quality control checks including FastQC, Illumina run specifications, and alignment quality control (INS-017 protocol). For each genotype, between 53 and 88 million read pairs were obtained. Genetic variants, including single nucleotide polymorphisms (SNPs) and insertions/deletions (InDels), were identified by aligning the reads to the CDC Frontier chickpea reference genome (assembly ASM33114v1; NCBI) with BWA-MEM v2.2.1 (Vasimuddin et al., 2019). Variants with a read depth <10 in at least one sample were excluded from downstream analysis to ensure data quality. The variant dataset generated in this study has been deposited into the European Variant Archive (EVA) at EMBL-EBI under the accession number PRJEB102951.

### Allelic variants calling and genomic distribution

Only homozygous allelic variants (SNPs and InDels) that were mapped to chromosomes and showed an alternative allele compared to the reference genome were selected for analysis. Specifically, variants were retained if they were present in both the simple leaf-type NIL (NH16/28-SL) and the corresponding parental line (CA2990), and differed from the reference genome (CDC Frontier, assembly ASM33114v1). The predicted impact of these variants was assessed using SnpEff v4.x (Cingolani et al., 2012), which classifies them into four categories based on their potential functional effect: modifier, low, moderate, or high impact. Variants located within annotated loci were further classified by genomic feature type, including protein-coding RNAs, small nuclear RNAs (snRNAs), small nucleolar RNAs (snoRNAs), and pseudogene transcripts. To identify potential candidate genes associated with the simple leaf phenotype, functional annotation of genes containing variants was performed using the NCBI gene database. Additionally, the chromosomal distribution and density of these variants were visualized using SRplot tools (Tang et al., 2023).

### Plant material, RNA isolation, cDNA synthesis and primer design

The most relevant MIQE guidelines for quantitative real-time PCR (qPCR) experiments (Bustin et al., 2009) were followed, in accordance with the practical recommendations of (Taylor et al., 2010). The expression profile of the candidate gene was analyzed in four genotypes (CA2990, WR315, NH16/28-SL, NH16/28-CL) and four cultivars (Dwelley, Sandford, CDC Frontier, Veleka) differing in leaf type (simple vs. compound) grown under greenhouse conditions. To account for biological variation in gene expression, three biological replicates were collected per genotype or cultivar. Young leaves were harvested, immediately frozen in liquid nitrogen, and stored at –80 °C until RNA isolation. The experimental design included a total of 24 samples (8 genotypes/varieties × 3 biological replicates). Total RNA was extracted using the Direct-zol RNA Kit (Zymo Research), directly from TRIzol-treated samples. RNA quality was assessed by spectrophotometry (NanoDrop) and electrophoretic integrity on agarose gel. First-strand cDNA synthesis was performed using the iScript cDNA Synthesis Kit (Bio-Rad, Hercules, CA), and samples were diluted to a working concentration of 20 ng/μl. To control for inter-run variation, a pooled cDNA sample representing all experimental conditions was included as an inter-run calibrator. No-template controls (NTCs) were also included to detect potential contamination. Specific primers targeting the *KNOX* gene (LOC101500499) were designed within the coding sequence (CDS), spanning an exon-exon junction using Geneious v.7.1.9. (https://www.geneious.com). The primers amplified a 143 bp fragment, had melting temperatures of 60 ± 1 °C, a GC content of 44%, and were 25 nucleotides long. The designed specific primers were qKNOX_F = 5’-GATTTGTTCAGCCAAAGTGAGCTTG-3’ and qKNOX_R = 5’-CTTAGCTCCCTAAGTTGTGAATGCA-3’. Primers were synthesized by Integrated DNA Technologies (Leuven, Belgium, www.IDTDNA.com).

### Quantitative real-time PCR assays

Quantitative real-time PCR (qPCR) was performed using the iTaq™ Universal SYBR^®^ Green Supermix (Bio-Rad, Hercules, CA, USA) on an ABI PRISM 7500 Real-Time PCR System (Applied Biosystems, Foster City, CA, USA). Each 10 μL reaction contained 5 μl of diluted cDNA (10 ng/μl), 5 μl of iTaq Universal SYBR Green Supermix (Bio-Rad, Hercules, California) and a primer pair with a concentration of 0.5 μM each. The thermal cycling conditions were as follows: initial denaturation at 95 °C for 10 minutes, followed by 40 cycles of 15 seconds at 95 °C and 1 minute at 60 °C. Specificity of amplification was confirmed by the presence of single, sharp peaks in melting curve analysis performed after completion of the amplification cycles. Fluorescence data were analyzed using 7500 Software v2.0.1, applying a uniform threshold value of 0.2 to determine quantification cycle (Cq) values for each gene-sample combination. The PCR efficiency of each primer pair was estimated using LinRegPCR v11.0 (Ruijter et al., 2009), based on raw normalized fluorescence (Rn) data. Relative gene expression (RGE) was calculated using the efficiency-corrected ΔCq method, incorporating multiple reference gene normalization and error propagation, as described by (Hellemans et al., 2007). The relative quantity (RQ) was calculated according to Equation 1:

(1) $$RQ=E^{\Delta Cq}$$

where *E* is the PCR efficiency for the corresponding primer pair, and *ΔCq* is the difference between the Cq values of the target gene (TG) and the geometric mean of the reference genes (RGs). Two reference genes, *PP2A* and *TFIIA*, previously validated as the most stable genes for qPCR normalization in chickpea, were used for normalization (Carmona-Molero et al., 2021; Caballo et al., 2019). The RGE values [Equation 2] were log-transformed and the significance values to determine differences of the expression of genes were obtained by the ANOVA test using the R programming language (R Development Core Team, 2025).

(2) RGE=RQTG/geomean[RQRG]

### Bioinformatic analysis and sequencing of *KNOX* gene

Descriptions and protein sequence identifiers (IDs) corresponding to potential candidate genes were retrieved from NCBI using the refseqR package (v.1.1.4; Die, 2024). These protein IDs were then used as queries for functional annotation analysis with the Gene Ontology (GO) tool Blast2GO, implemented in the OmicsBox platform (v3.0.27), to assign GO terms to each sequence (Götz et al., 2008). To predict intron–exon boundaries, determine the position of the allelic variant associated with leaf type, and assist in primer design, the mRNA (XM_004512716.4) and protein (XP_004512773.1) sequences of the *C. arietinum homeobox protein knotted-1* (*KNOX*) were aligned using Geneious v7.1.9 (https://www.geneious.com). To validate the presence of the allelic variant distinguishing simple and compound leaf types, a primer pair was designed to amplify the target region. (KNOX_F: 5’-CCACGTATGCATGCAAGGGT-3’ and KNOX_R: 5’-CCAAGTGAGCTTCAACAAGAAATTGA-3’). Genomic DNA was extracted from three genotypes or cultivar with simple leaves (CA2990, Dwelley and Sandford) and three with compound leaves (WR315, CDC Frontier and Veleka). PCR amplifications were performed in 25 μL reaction volumes containing 5 μl DNA of each sample, 5 μl of 5x reaction buffer (comprises 5 mM dNTPs and 15 mM MgCl_2_), 10 μM of each primer and 0.4 U MyTaq DNA polymerase Bioline. The expected amplicon length was 394 bp. PCR products from the six chickpea genotypes were purified using the Exo-CIP Rapid PCR Cleanup Kit (New England Biolabs, USA), following the manufacturer’s protocol. Purified PCR products were bidirectionally sequenced (both sense and antisense strands) via Sanger sequencing at StabVida (Caparica, Portugal). For each sample, forward and reverse reads were aligned to generate a consensus sequence, and allelic variants were identified using multiple alignment tool from Geneious.

### Allele-specific diagnostic marker

A KASP by Design (KBD) assay (LGC Genomics, Hoddesdon, UK) was developed to distinguish between simple and compound leaf types in chickpea, based on an allele-specific variant detected in the resequencing of the parental lines and NILs. This allelic variant, a single-base deletion located at position 413 bp of the *KNOX* mRNA sequence, was used to design the KASP_KNOX marker. The following primers were used for the assay: Allele_X = 5’-CTACTCAAATATAGATATGGAATCTAAGAGG-3’, Allele_Y = 5’-TCTACTCAAATATAGATATGGAATCTAAGAGA-3’, and the conserved primer Common = TTGATGATGGTGATAACAAGTACAAAGAAC. To validate the KASP marker, 77 RILs and the two parental lines (CA2990 and WR315) from the RIP16 population were genotyped. Genomic DNA was extracted using the DNeasy Plant Mini Kit (Qiagen, Valencia, CA, USA). Each 10 μL KASP reaction contained 5 μL of DNA (20 ng/μL), 5 μL of 2x KASP Master Mix and 0.14 μL KASP Assay Mix (allele-specific primer and common primer). Genotyping was performed on a BIO-RAD CFX96 Real-Time PCR System using the following thermal cycling conditions: 94 °C for 15 min, followed by 10 cycles of 94 °C for 20 s, 61 °C-55 °C for 60 s by 0.6 °C decrease per cycle, then by 36 cycles of 94 °C for 30 s and 55 °C for 30 s, and finally at 37 °C for 30 s. Genotyping results were visualized using the “ggplot2” package in R (Wickham, 2016).

### Statistical data analysis

Segregation data for leaf type in the RIL population were analyzed for goodness of fit to the expected 1:1 ratio using the chi-square test. A *t*-test (*P* < 0.05) was performed to determine whether there was a significant difference in mean leaf surface area between leaf type groups. To assess the relationship between the KNOX marker and the phenotypic traits (leaf type and leaf surface), Pearson correlation coefficient analysis was performed using the Performance Analytics R package (Peterson and Carl, 2025). In addition, a linear regression analysis was conducted using Statistix 8.0, with leaf type as the dependent variable and the KNOX marker as the independent variable, to estimate the coefficient of determination (R²), representing the percentage of phenotypic variation explained by the marker.

### Identification, phylogenetic analysis, and conserved domain characterization of *KNOX* genes

To explore the phylogenetic relationships of the chickpea *KNOX1* gene with the homologous sequences in legumes, BLASTP searches were first performed using the *C. arietinum KNOX* protein sequence (XP_004512773.1) as a query against eight related legume species, along with *Arabidopsis thaliana*. The retrieved amino acid sequences were aligned with ClustalW and used to construct a phylogenetic tree. Next, the same query was used against the *C. arietinum* genome itself to identify all *KNOX* gene copies present in chickpea. For each chickpea *KNOX* sequence identified, putative orthologs in *Medicago truncatula* and *A. thaliana* were retrieved by BLASTP searches. These sequences were then aligned using ClustalW (Higgins et al., 1994) and subjected to phylogenetic analysis to classify the *C. arietinum KNOX* proteins. Bootstrap consensus trees were constructed using the Maximum Likelihood method (Jones et al., 1992) with 1,000 replicates to represent the evolutionary history of the analyzed taxa (Felsenstein, 1985). Evolutionary distances were calculated using the Poisson correction and are expressed as the number of amino acid substitutions per site (Zuckerkandl and Pauling, 1965). Positions containing gaps or missing data were excluded. All evolutionary analyses were performed in MEGA11 software (Tamura et al., 2021).

Homology analysis and multiple sequence comparison of KNOX proteins from *C. arietinum*, *M. truncatula* and *A. thaliana* were performed using Geneious v7.1.9 (https://www.geneious.com). Conserved domains of each KNOX protein were predicted by comparison against the NCBI Conserved Domain Database (CDD v3.21; (https://www.ncbi.nlm.nih.gov/Structure/cdd/wrpsb.cgi) using default parameters.

## Results

### Phenotypic data analysis

**Figure 1:**
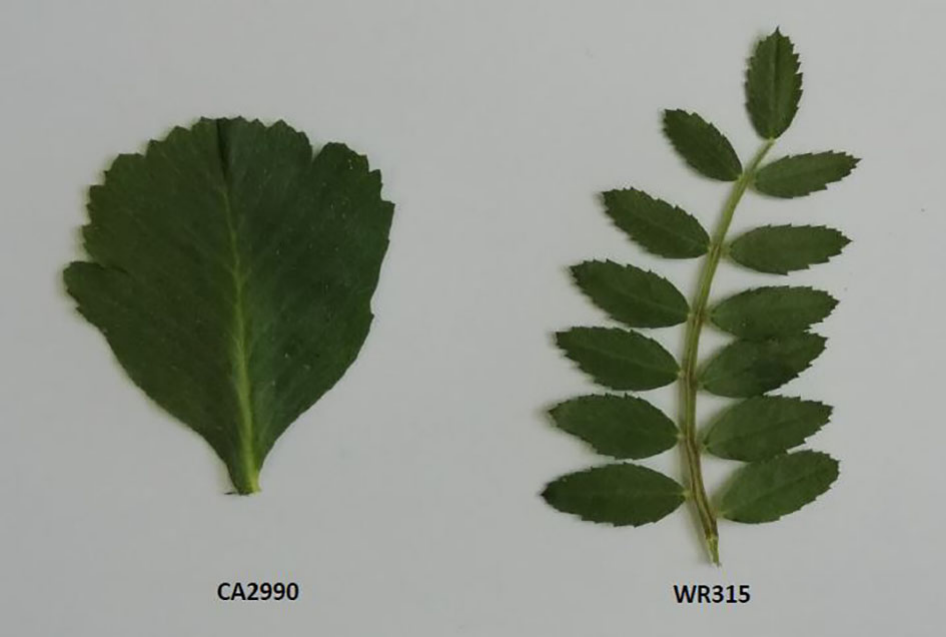
Differential leaf morphologies of parental lines CA2990 (simple) and WR315 (compound).

**Figure 2:**
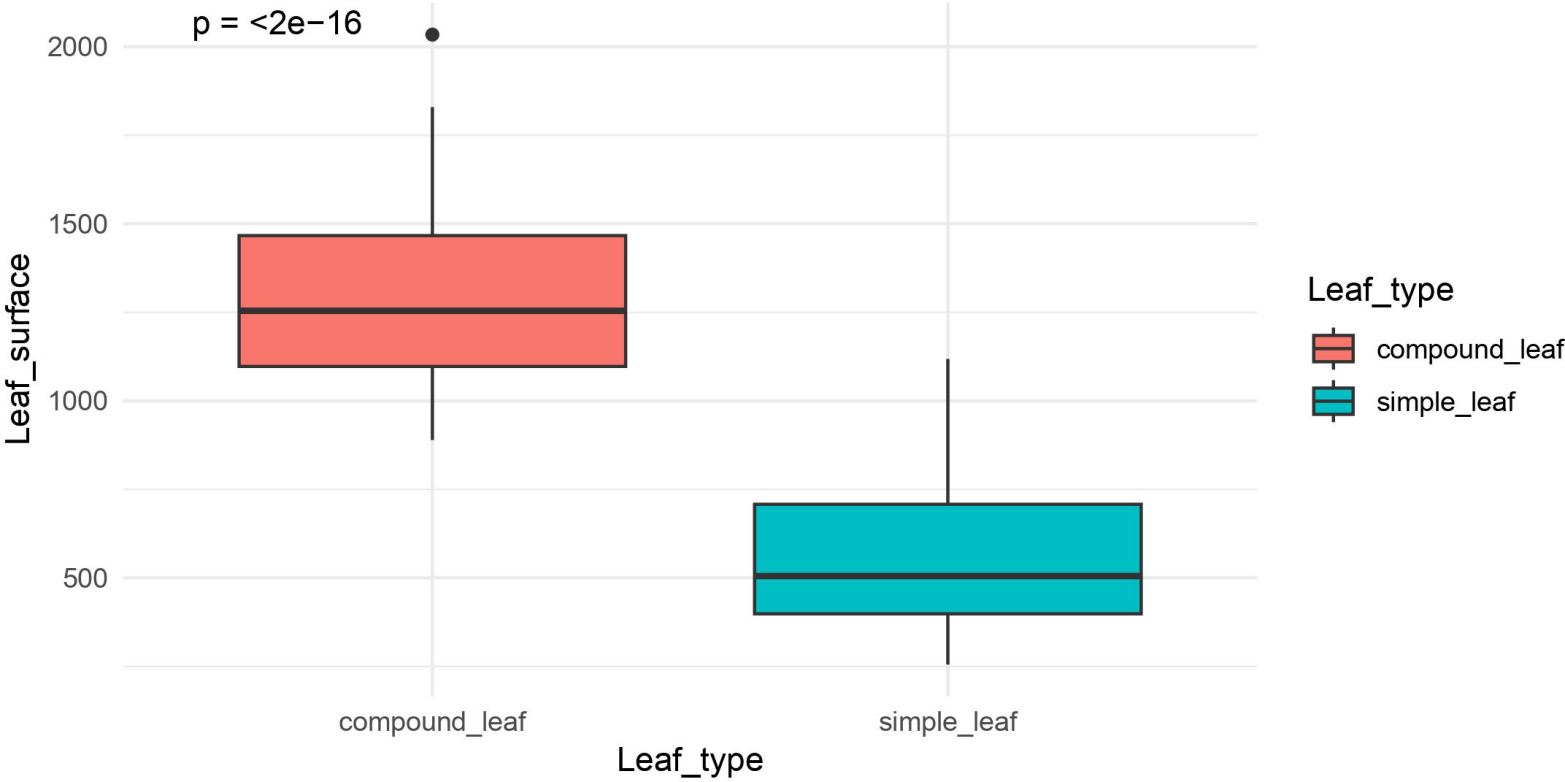
Boxplot and *t*-test analysis showing significant differences in leaf surface area between leaf morphology groups.

The leaf morphology of the parental lines used in this study—CA2990 (simple leaf) and WR315 (compound leaf)—is shown in **Figure 1**. Among the 77 F_6:7_ plants from the CA2990 × WR315 population evaluated for leaf type, 34 exhibited simple leaves, 41 had compound leaves, and 2 were heterozygous. The observed segregation conformed to the expected 1:1 ratio for a single-gene model (χ² = 0.42, *P* = 0.65). Leaf surface area ranged from 255.33 to 1,118.4 mm² in plants with simple leaves and from 889.36 to 2,034.10 mm² in those with compound leaves. Some overlap in leaf surface area was observed, with a few simple-leaf lines showing high values and some compound-leaf lines showing low values. A *t*-test revealed a significant difference between the mean leaf surface areas of the two groups (**Figure 2**).

### Allelic variants calling and genomic distribution

Sequencing data revealed up to 393,670,329 positions read, with a total of 865,854 variant calls. Of these, 76% were SNPs and 24% were InDels relative to the chickpea reference genome (ASM33114v1). Among the samples, WR315 and NH16/28-CL (both with compound leaf phenotypes) exhibited the highest proportion of variant alleles, at 69.5% and 60.7%, respectively. In contrast, CA2990 and NH16/28-SL (both with simple leaf phenotypes) showed lower proportions, each below 60% (**Supplementary Table 1**). The proportion of SNPs and InDels shared between samples was 32.5%. A low number of heterozygous positions was detected in each sample, corresponding to an average observed heterozygosity of 0.02%, consistent with the expected residual heterozygosity predicted by Mendelian genetics for highly self-fertilizing species (Perez-Rial et al., 2024).

**Figure 3:**
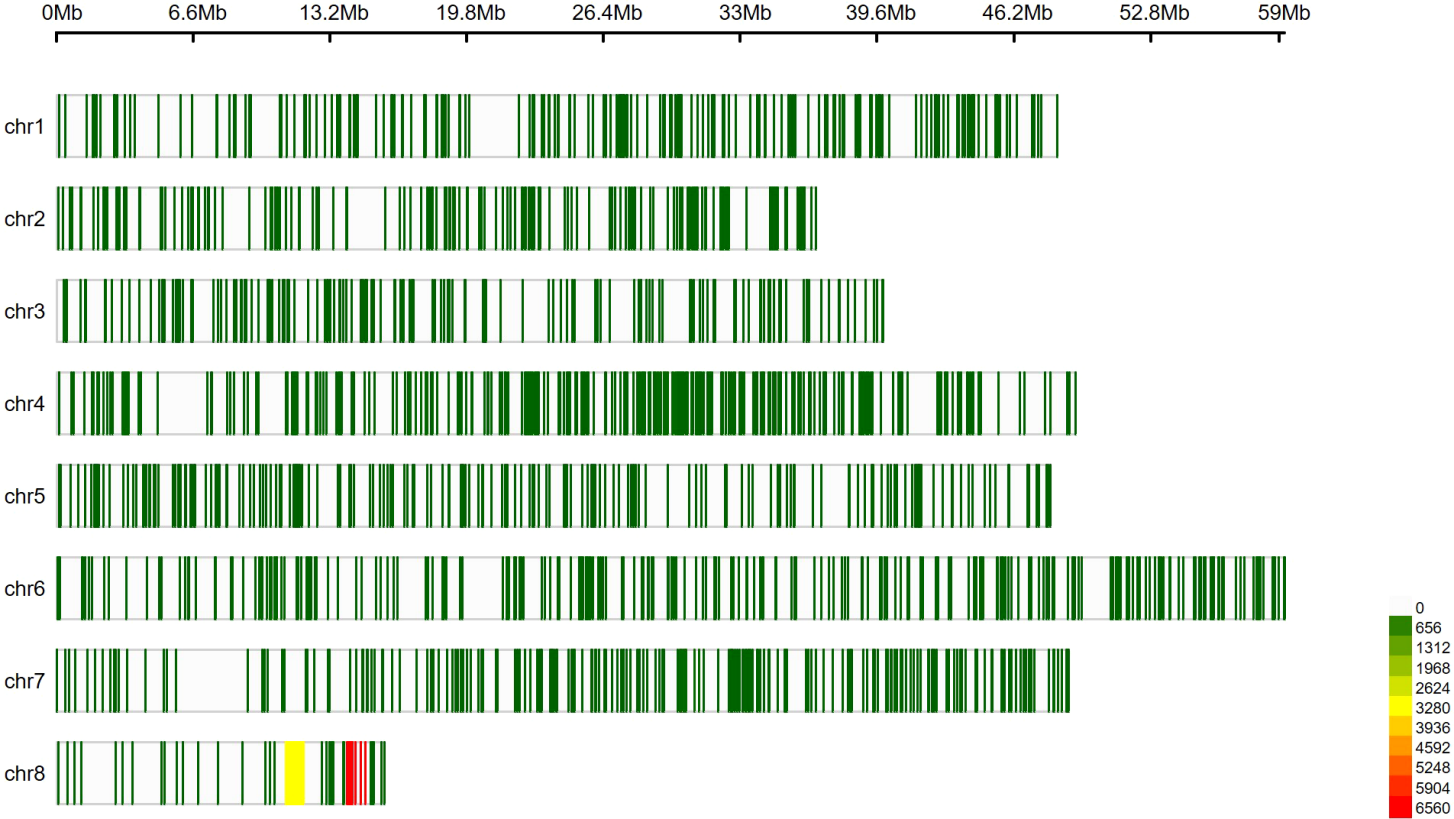
Density map and genomic distribution of allelic variants across the eight chromosomes of chickpea, using a 1 Mbp window size. Green indicates regions of lowest variant density, while red represents regions of highest density. Pearson correlations and histograms showing the distribution of the KNOX marker and the phenotypic traits (leaf type and leaf surface area). The frequency distributions are displayed along the main diagonal as histograms. Scatter plots for each pair of traits are shown below the diagonal, and the corresponding Pearson correlation coefficients (r) are presented above the diagonal. The red line represents the regression slope. The x- and yaxes show the measured values. Significance level is indicated by *** for p < 0.001.

Comparison of sequencing data from two samples with a compound leaf phenotype and two with a simple leaf phenotype revealed a total of 19,889 variants, of which 79.2% were successfully mapped to chromosomes. **Figure 3** illustrates the density map of the genomic distribution of these variants across the eight chickpea chromosomes. Variant counts per chromosome ranged from 610 on chromosome 5 to 9,999 on chromosome 8, covering a total genomic length of 345.8 Mbp. The distribution of allelic variants per 1 Mbp was generally proportional to chromosome size (ranging from 11 to 20 variants/Mbp), except for chromosome 4, which showed a higher density of 33 variants/Mbp. The highest average variant density was observed on chromosome 8, with 632 variants/Mbp (**Table 1**).

**Table 1:** Chromosome-wise distribution, number of variants, and genomic coverage in parental and near-Isogenic lines used in this study.

Chromosome	ID_chr.	Lenght (Mb)	Variants number	Variants density
Ca1	NC_021160.1	48.27	611	13
Ca2	NC_021161.1	36.62	585	16
Ca3	NC_021162.1	39.88	450	11
Ca4	NC_021163.1	49.17	1607	33
Ca5	NC_021164.1	47.94	610	13
Ca6	NC_021165.1	59.26	945	16
Ca7	NC_021166.1	48.85	955	20
Ca8	NC_021167.1	15.82	9999	632
TOTAL		345.8	15762	

### Functional annotation of candidate genes

In a first attempt to identify potential candidate genes responsible for leaf type, only homozygous allelic variants that were assigned to chromosomes and showed an alternative allele in the parental line and NIL with simple leaf (CA2990 and NH16/28-SL) compared to the reference genome were selected for theoretical impact analysis. This selection reduced the number of allelic variants to 464, with 100 located on chromosome 6 and 125 on chromosome 8. Of these variants, 436 were annotated as having a theoretical impact categorized as modifier, two as moderate, one as high impact, and 25 could not be categorized.

Functional annotation was performed for genes containing allelic variants annotated as moderate (LOC101493281 and LOC101499849) or high impact (LOC101500499), all classified as protein-coding RNAs. These three genes are located on chromosome 8 between positions 11.59 and 11.83 Mb (spanning a 240 kb region). LOC101493281 was annotated as an uncharacterized gene, whereas LOC101499849 was described as a “glutamine synthetase nodule isozyme”. Both genes harbored missense variants, in which a single nucleotide change results in the incorporation of a different amino acid into the protein. None of the annotations suggested a likely role in determining leaf type. The high-impact allelic variant corresponded to LOC101500499, which is functionally annotated as a “homeotic protein knotted-1-like (KNOX).” This sequence is associated with Gene Ontology (GO) terms across three categories: nucleus (GO:0005634; cellular component), DNA binding (GO:0003677; molecular function), and regulation of DNA-templated transcription (GO:0006355; biological process). The variant is a frameshift caused by a single-nucleotide deletion, altering the translational reading frame and potentially resulting in mutation, protein truncation, or loss of function. Given the well-documented role of *KNOX* genes in regulating leaf development and the type of allelic variant observed, the *KNOX* gene (LOC101500499) emerged as a strong candidate responsible for the development of compound leaves.

### Expression analysis of *KNOX* gene

To confirm whether the mutation in the *KNOX* gene is responsible for simple leaf development, RT-qPCR analysis was performed on young leaves from eight genotypes differing in leaf type phenotype. To verify the sensitivity and specificity of the assay, the primer pair qKNOX Fw/Rv was designed to span an exon–exon junction (from exon 2 to exon 3), allowing the detection of potential genomic DNA (gDNA) contamination in the cDNA samples. The primers pair amplified a 3,087 bp product when using gDNA as the template and a 143 bp fragment when using cDNA. None of the cDNA samples produced bands corresponding to residual gDNA, confirming that they were free of gDNA contamination. Dissociation curve analysis further confirmed that the qKNOX Fw/Rv primer pair produced a single, specific PCR product (77.76 ± 0.24 °C, mean ± SD). The mean PCR efficiency was 95.4% for KNOX, and 98.9% and 99.3% for the reference genes PP2A and TFIIA, respectively.

**Figure 4:**
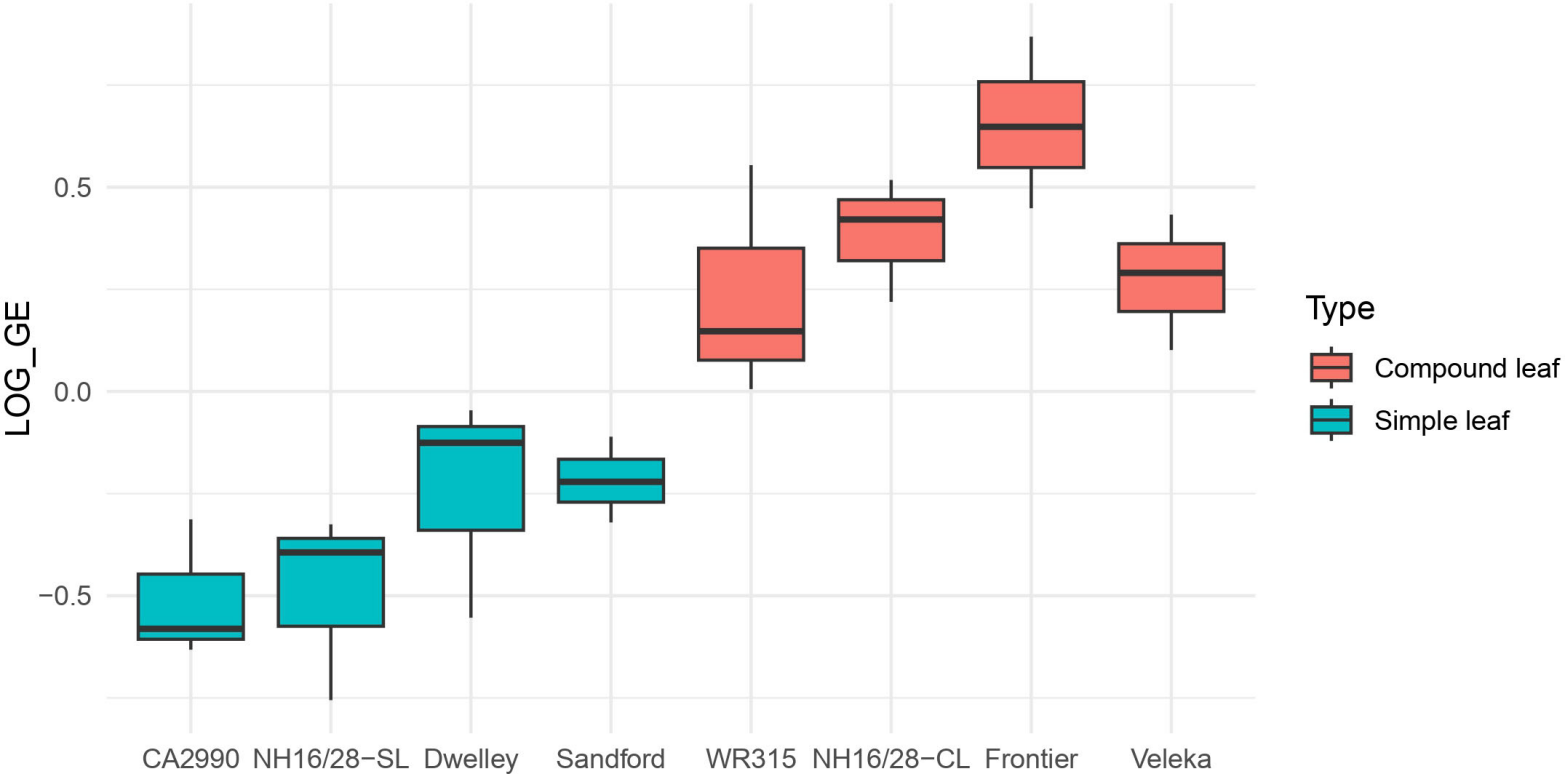
Boxplot showing transcript levels of *KNOX* gene in four simple leaf genotypes and four compound leaf genotypes. Expression values were log-transformed, and a statistically significant difference was observed between the two groups (p < 0.001).

The overall expression level of the *KNOX* gene was significantly lower (p < 0.001) in genotypes with simple leaves compared with those with compound leaves (**Table 2** and **Figure 4**). These highly significant differences in *KNOX* transcript abundance between genotypes suggest a direct relationship between the identified mutation and a loss of gene function, which is reflected in the reduced expression levels observed in simple-leaf genotypes. This finding supports the hypothesis that the KNOX mutation underlies the simple leaf phenotype.

**Table 2:** Gene expression of the *KNOX* gene.

ID_Sample	Phenotype	Type	Log_average_expression
CA2990	Simple leaf	Genotype	-0.509
NH16/28-SL	Simple leaf	Genotype	-0.492
Dwelley	Simple leaf	Cultivar	-0.242
Sandford	Simple leaf	Cultivar	-0.217
WR315	Compound leaf	Genotype	0.236
NH16/28-CL	Compound leaf	Genotype	0.386
CDC Frontier	Compound leaf	Cultivar	0.655
Veleka	Compound leaf	Cultivar	0.275

Values indicate log average expression ratios of three biological replicates from each sample. All the samples showed statistically significant differences in regulation (p < 0.001).

### Bioinformatics analysis of *KNOX* gene

LOC101500499 (Ca8: 11,832,784 - 11,837,552) was annotated as *homeobox protein knotted-1* (KNOX), a homologue of the *KNAT2* gene in *A. thaliana* and the Fused Compound Leaf1 (FCL1) in *M. truncatula*. The chickpea *KNOX* gene consists of three exons and two introns, with a total length of 4,741 nucleotides. The corresponding mRNA sequence (XM_004512716.4) is 1,041 nucleotides long, and the coding DNA sequence (CDS) spans positions 400 to 882 bp. The 482 bp open reading frame (ORF) encodes a predicted protein of 160 amino acids, containing both the KNOX1 and KNOX2 domains. The alignment of mRNA and CDS sequences for compound- and simple-leaf types, together with the positions of the two primer pairs designed in this study and the corresponding translation, is shown in **Supplementary Figure 1**.

**Figure 5:**
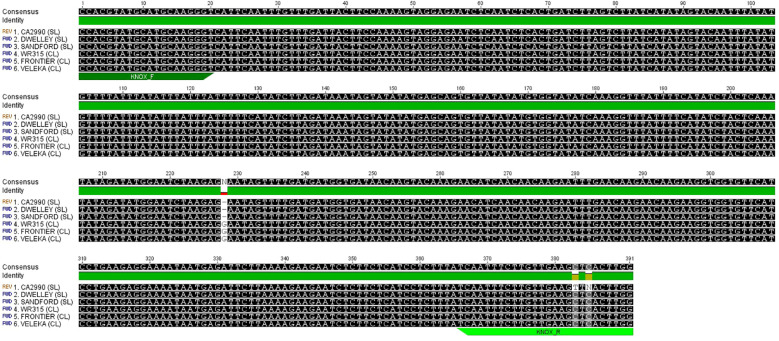
Alignment of the DNA nucleotide sequences from the six genotypes differing to leaf type phenotype. The position of the primer pair, as well as the deletion found are indicated.

**Figure 6:**
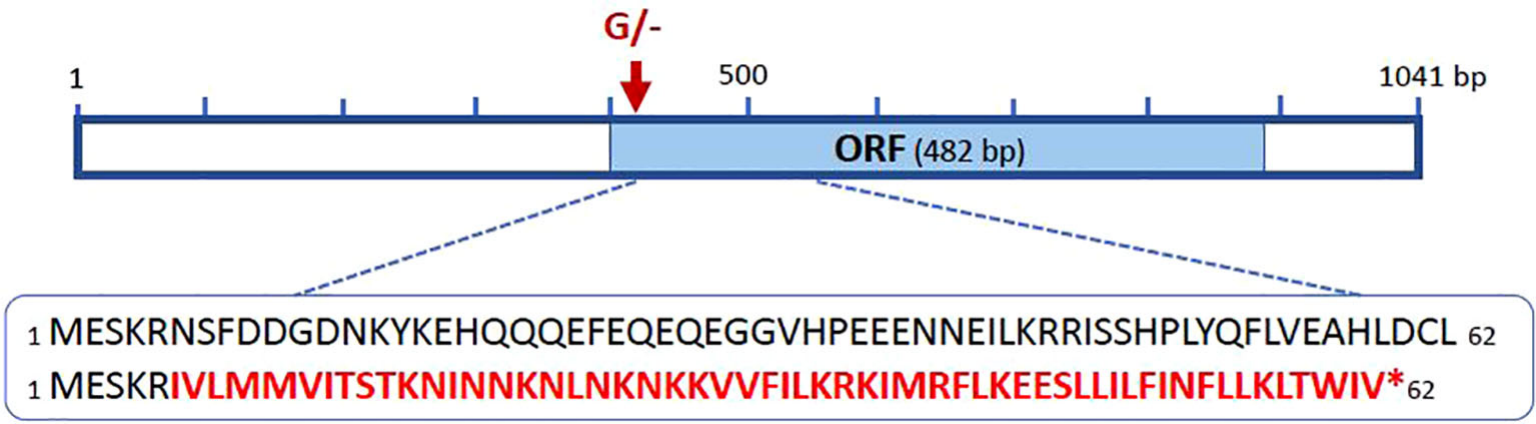
Genomic organization of the *KNOX* gene. The mRNA sequence and open reading frame (ORF) are represented as solid boxes. The allelic variant (single-nucleotide deletion) distinguishing compound and simple leaf types is indicated by an inverted arrow. This deletion, located within the ORF, causes a frameshift that alters the amino acid sequence and introduces a premature stop codon (*) in the simple-leaf genotypes.

The mRNA transcript sequence was used to locate and validate the single-nucleotide deletion detected by WG-Seq that distinguishes simple and compound leaf types. A primer pair was designed to amplify and sequence the corresponding DNA region using the same genotypes analyzed for the gene expression assay, excluding the NIL samples (**Figure 5**). Alignment of the nucleotide sequences revealed that all genotypes were identical, except for a single-nucleotide deletion present in the simple-leaf genotypes, confirming the allelic variant previously identified in the NIL lines by WG-Seq. This deletion (G/–) was located 14 bp downstream of the ORF start, resulting in a frameshift that introduced a premature stop codon in the simple-leaf genotypes (**Figure 6**). This finding also supports that the mutation identified in the *KNOX* coding sequence underlies the simple-leaf phenotype.

### KNOX diagnostic marker

**Figure 7:**
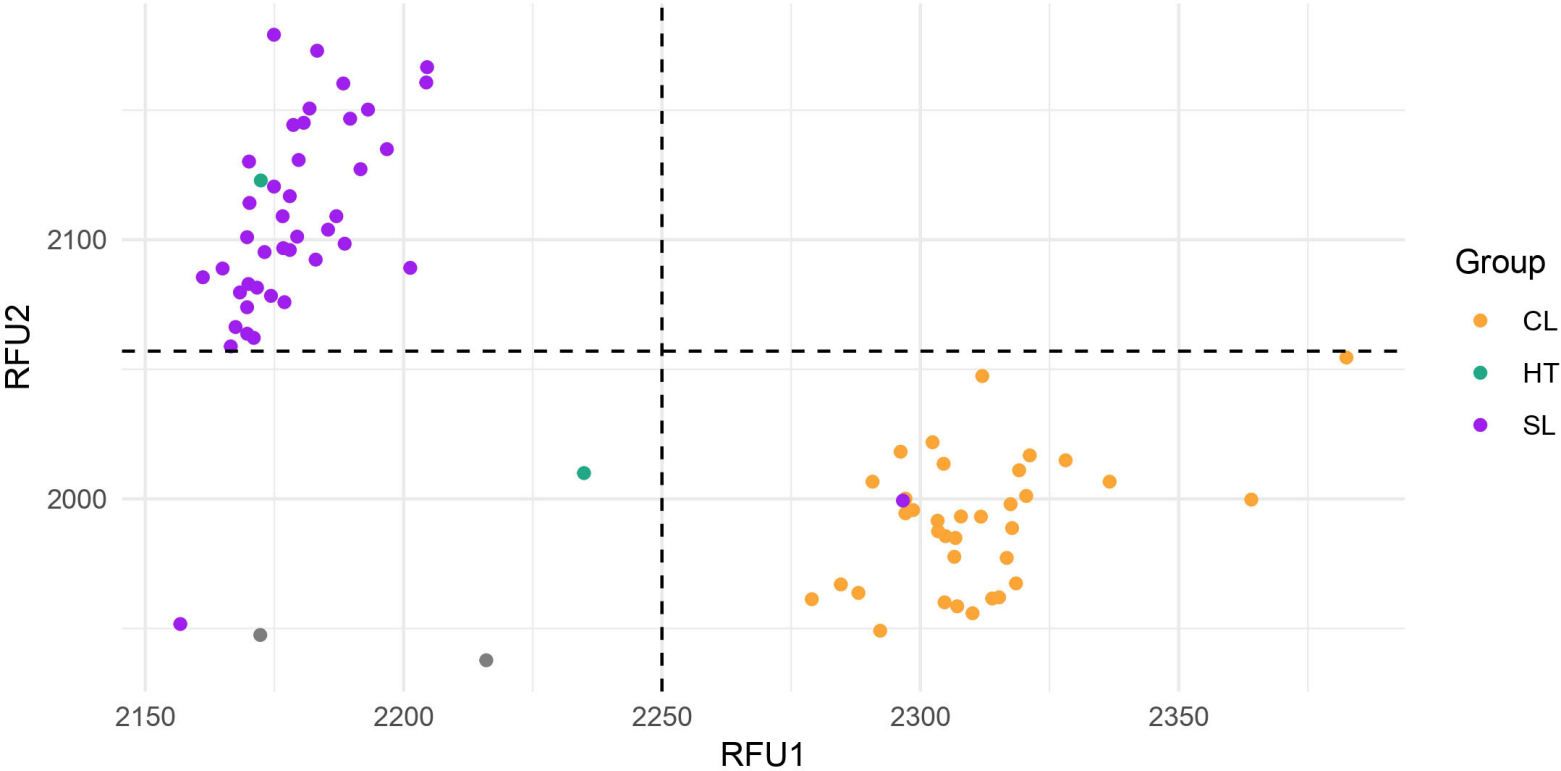
KASP genotyping results for the KNOX marker in 77 individuals from the RIL population. The scatterplot shows allelic discrimination along the x- and y-axes, with accessions clustering into three genotype groups: FAM homozygotes (purple), HEX homozygotes (orange), and FAM/HEX heterozygotes (HT, green). No-template controls (NTC) are shown in grey. Allele “G” (orange) co-segregates with the compound-leaf phenotype (CL), whereas allele “–” (purple) corresponds to the mutant simple-leaf phenotype (SL).

**Figure 8:**
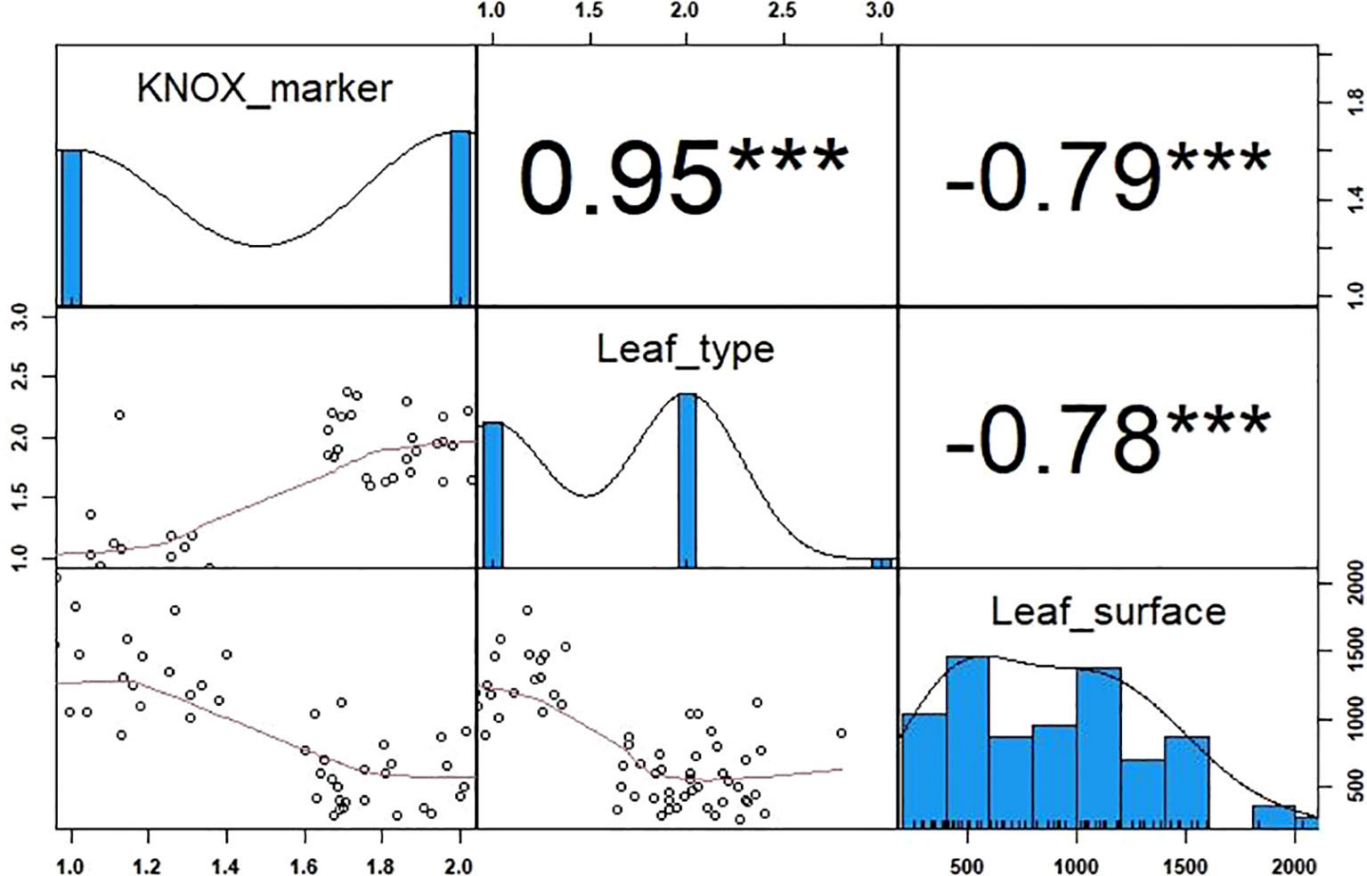
Pearson correlations and histograms showing the distribution of the KNOX marker and the phenotypic traits (leaf type and leaf surface area). The frequency distributions are displayed along the main diagonal as histograms. Scatter plots for each pair of traits are shown below the diagonal, and the corresponding Pearson correlation coefficients (r) are presented above the diagonal. The red line represents the regression slope. The x- and y-axes show the measured values. Significance levels are indicated by *, **, and *** for p < 0.05, p < 0.01, and p < 0.001, respectively.

The single-base deletion detected on the simple leaf phenotypes was used to design a Kompetitive Allele Specific PCR (KASP) marker. The parental lines and the RIL population were used to validate KNOX marker. The genotypic data associated with each allele (G vs. –) showed a clear separation between individuals with compound and simple leaf types (**Figure 7**). As expected, Pearson’s correlation between the KNOX marker and leaf type was strong and highly significant (r = 0.95), while leaf surface area showed a strong negative correlation (r = –0.79; **Figure 8**). The marker explained 90% of the phenotypic variation for leaf type (R² = 0.90, p < 0.001), indicating a strong genetic association between the marker and the trait, and suggesting that the mutation is located in close proximity to, or within, the causal gene.

### Phylogenetic analysis, and conserved domain characterization

**Figure 9:**
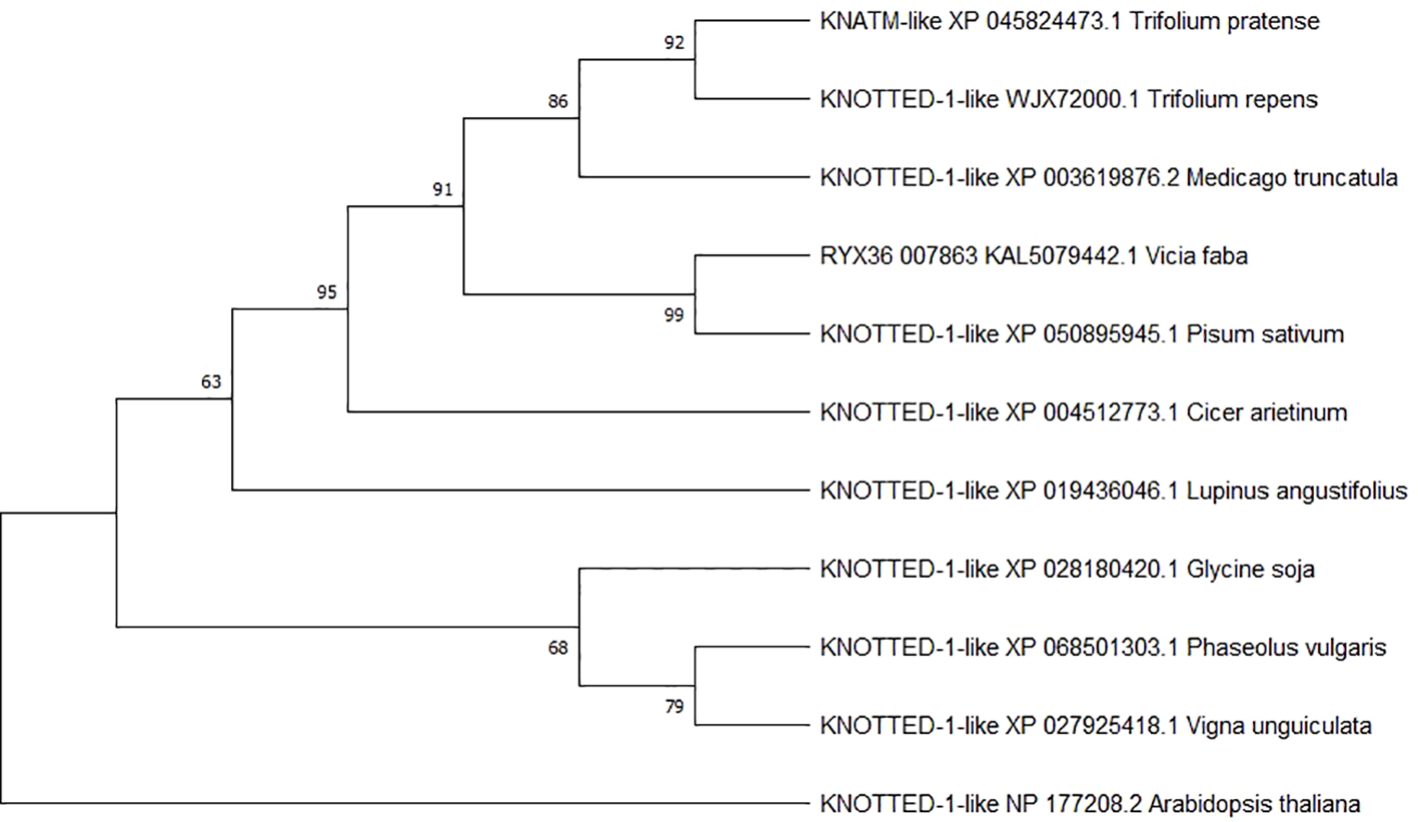
Phylogenetic tree of KNOTTED proteins from chickpea (*Cicer arietinum* L.) and other plant species. Numbers on nodes represent bootstrap values. Evolutionary analyses were performed using MEGA11.

The phylogenetic tree based on amino acid sequences of chickpea *KNOTTED-1-like* (XP_004512773.1) and its orthologs revealed two well-defined clusters, separating legume species from *Arabidopsis* and supporting the close phylogenetic relationships among Fabaceae sequences compared with Brassicaceae. Based on percent identity of the KNOTTED sequences, the legumes were further divided into two major clades: Galegoid species (*Trifolium pratense*, *Trifolium repens*, *M. truncatula*, *Vicia faba*, *Pisum sativum*, *C. arietinum*, and *Lupinus angustifolius*) and Phaseoloid species (*Glycine soja*, *Phaseolus vulgaris*, and *Vigna unguiculata*). The chickpea KNOTTED sequence, belonging to the tribe Cicerae, showed the closest relationship with *V. faba* and *P. sativum*, both of which cluster within the tribe Fabaeae. In contrast, *Trifolium* and *Medicago*, belonging to the Trifoliae, formed a separate cluster (**Figure 9**).

**Figure 10:**
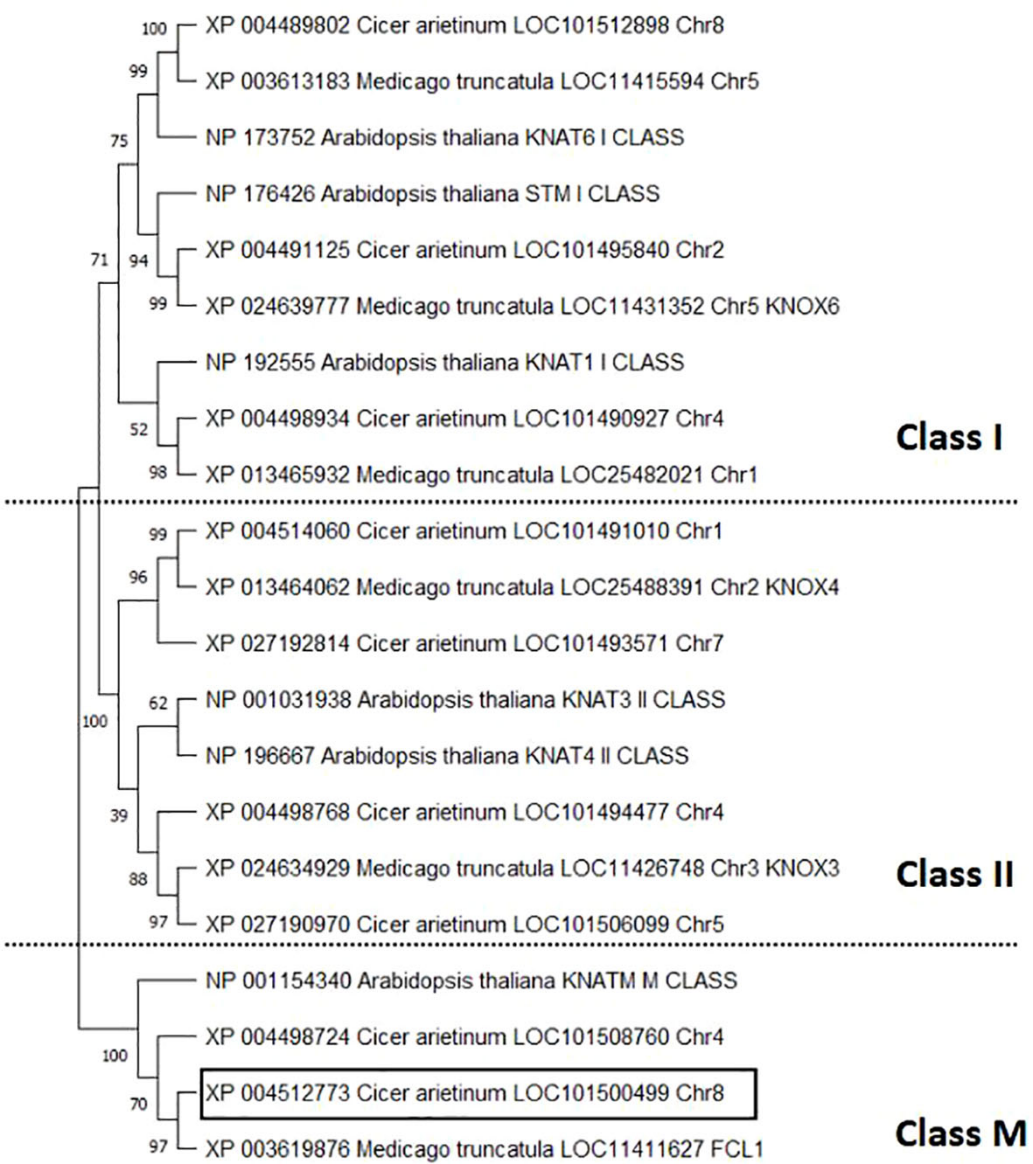
Categorization of the KNOX gene family of chickpea by phylogenetic tree analysis of sequences from *Cicer arietinum*, *Medicago truncatula* and *Arabidopsis thaliana*. Numbers on nodes represent bootstrap values.

In the chickpea reference genome, a total of nine *KNOX* genes were identified, distributed across all chromosomes except chr3 and chr6. Using the amino acid sequences, a phylogenetic tree of 21 sequences from three species was constructed to analyze the evolutionary relationships among *KNOX* genes family members in *C. arietinum*, *M. truncatula* and *A. thaliana* (**Figure 10**). Chickpea KNOX proteins were categorized into three classes: Class I, Class II and Class M, encompassing 3, 4 and 2 members respectively. Within Class I KNOX proteins, the chickpea KNOX sequences grouped into three subgroups homologous to KNAT1, STM and KNAT6 from *A. thaliana*, consistent with the characterization of the KNOX gene family in *Medicago* species (Luo et al., 2025). The four chickpea sequences classified as Class II were highly similar to KNOX4 and KNOX3 from *M. truncatula*. Class M comprises two chickpea KNOX sequences, including the sequence under study (LOC101500499), which showed high bootstrap support value to FCL1 from *M. truncatula* and *KNATM* gene from *A. thaliana*. This suggests that *KNOX* sequences are very closely related between species.

The conserved domains of the *KNOX* gene family in *C. arietinum* were analyzed in the context of inter-species evolutionary relationships (**Supplementary Figure 2**). Members of Class I and Class II were found to possess four conserved structural domains: KNOX1, KNOX2, ELK, and Homeobox KN. In contrast, Class M *KNOX* genes contained only the MEINOX domain (KNOX1 and KNOX2) and lacked the ELK and Homeobox KN domains.

## Discussion

Plant leaves serve as the primary photosynthetic organs, converting carbon dioxide and water into carbohydrates and oxygen through the capture of solar energy. Beyond their central role in photosynthesis, leaves also act as key sensory organs that perceive environmental cues and respond through morphological and physiological adjustments. Thus, among the most extensively studied agronomic traits associated with leaf development and variation are photosynthetic efficiency and drought tolerance. Leaves originate from the peripheral region of the shoot apical meristem and display remarkable diversity in shape and size. Traditionally, they are classified into two major morphogenetic types: undivided simple leaves and compound leaves composed of multiple leaflets. The development and diversification of leaf forms have long been central topics in plant developmental biology and evolutionary studies (Champagne et al., 2007).

The diverse leaf morphologies observed in legumes not only provide valuable systems for studying the mechanisms of leaf development but also represent important traits for breeding programs aimed at improving agronomic performance (Luo et al., 2025). *Medicago* serves as an excellent model for investigating compound leaf development, owing to its extensive genomic resources and the ease with which both forward and reverse genetic approaches can be applied. However, the compound trifoliate leaf of *Medicago* is relatively simple in structure compared with the more complex leaf architecture found in *C. arietinum*. The broader geographical distribution and ecological adaptability of chickpea suggest the involvement of additional regulatory factors that define its unique leaf morphometric and anatomical features (Basu and Parida, 2023). Although these variations have likely contributed to the species successful adaptation to diverse environmental conditions, comprehensive studies addressing the developmental and evolutionary bases of leaf variation in chickpea are still largely absent, leaving a significant gap in our understanding of leaf morphogenesis within this important legume species.

### The chickpea homeotic protein knotted-1-like gene

In this study, we employed a combination of genetic and computational approaches, including the development of segregating plant populations and high-throughput sequencing data, to investigate the molecular mechanisms underlying compound leaf formation in *C. arietinum*. Near-isogenic lines represent well-defined plant materials that restrict genetic variation to targeted genomic regions, thereby providing a robust resource for genetic and functional analyses in chickpea. Our group has successfully developed NIL pairs for studies on growth habit (Ali et al., 2015), double/single pod formation (Ali et al., 2016; Caballo et al., 2022), nodulation (Ali et al., 2014), Fusarium wilt resistance (Castro et al., 2010; Caballo et al., 2019; Jendoubi et al., 2016), and flowering time (Perez-Rial et al., 2024). The NILs developed in this study further enrich these resources, offering valuable plant materials for future genetic and functional research in chickpea. Our sequencing analyses identified LOC101500499, located on chromosome 8 and functionally annotated as a *homeotic protein knotted-1-like* (*KNOX*) gene, as a strong candidate underlying this trait. Detailed bioinformatic analysis revealed a single-nucleotide deletion (G/–) located 14 bp downstream of the start of the open reading frame (ORF). To test whether this mutation in the coding sequence of the *KNOX* gene plays a determining role in the simple leaf phenotype, we analyzed the transcriptomic profiles of several chickpea genotypes exhibiting contrasting leaf morphologies (compound vs. simple leaves). Significant differences in *KNOX* transcript levels between the two groups support the hypothesis that this mutation plays a key role in determining the simple leaf phenotype in chickpea. After identifying the candidate genetic variants (G/–), we aimed to confirm whether the variant was consistently associated with the particular trait, so we designed a KASP marker to validate and genotype these variants across a larger set of individuals or populations (Semagn et al., 2014). The marker explained 90% of the phenotypic variation in leaf type, providing further evidence for the determining role of the *knotted-1-like* gene in regulating leaf morphology in chickpea.

### The inverted repeat-lacking clade

Among the key regulators of leaf morphology, the well-known *KNOX* genes were first identified more than two decades ago (Tsuda and Hake, 2015; Furumizu et al., 2015; Hake et al., 2004). Notably, the *KNOX* gene family is characterized by its broad distribution and high degree of conservation across plant species. Members of this family play pivotal roles in plant development, particularly in the regulation of compound leaf formation and growth (Wang et al., 2021b, 2021a). Although identifying the chickpea *homeotic protein knotted-1-like* gene (LOC101500499) as a key factor in compound leaf development was an important achievement, it was also an intriguing result, since *KNOX1* gene expression has been reported to be completely absent from compound leaves in members of the inverted repeat-lacking clade (IRLC) (Champagne et al., 2007; Basu and Parida, 2023; Liu et al., 2023). The IRLC is a large subclade of papilionoid legumes that happens to be marked by loss of one copy of the 25-kb inverted repeat in the chloroplast genome (Wojciechowski et al., 2004). Although *KNOX1* genes play pivotal roles in compound leaf development across all non-IRLC legumes (Champagne et al., 2007), in IRLC members—such as *P. sativum* (pea) and *M. truncatula*—the function of *KNOX1* genes is completely replaced by the floral meristem identity genes *FLORICAULA* (*FLO*) and the *LEAFY* (*LFY*) orthologues *UNIFOLIATA* (*UNI*) and *SINGLE LEAFLET1* (*SGL1*) (Champagne et al., 2007; Wang et al., 2008; Hofer et al., 1997; He et al., 2020). Consequently, it has been emphasized that *KNOX1* is not always involved in compound leaf development (Nakayama et al., 2022). However, our findings clearly support a role for the chickpea *KNOX1* gene in the compound leaf development, representing an unexpected result given that chickpea is a member of the IRLC clade. Therefore, our data required further investigation.

### The *KNOX1* chickpea gene is not a class I *KNOX* gene

We identified a total of nine *KNOX* genes distributed across all chickpea chromosomes, except for chromosomes 3 and 6. The same number of *KNOX* genes reported in *M. truncatula* (Luo et al., 2025), supporting the high degree of conservation between these two legume species previously demonstrated through synteny analyses (Parween et al., 2015; Gupta et al., 2017). This conservation pattern is consistent with that observed for other gene families in legumes (Carmona-Molero et al., 2021; Die et al., 2018). Through phylogenetic analysis, the *KNOX* gene family in *C. arietinum* was classified into three subclasses. Each subclass contained genes homologous to those identified in *A. thaliana* and *M. truncatula*. Together with class I and class II sequences, the class M proteins formed a distinct clade. Interestingly, LOC101500499 was grouped within the M subclass, exhibiting high amino acid sequence identity with FCL1 from *Medicago* and KNATM from *Arabidopsis*.

Proteins belonging to the M subfamily within the *KNOX* gene family are thought to have arisen later in plant evolution and appear to be restricted to eudicots. These proteins likely originated from a lineage of class I or class II *KNOX* genes that lost their ELK and homeodomain (HD) regions (Luo et al., 2025; Gao et al., 2015). The functional diversification of *KNOX* genes is likely attributable to differences in the number and composition of conserved structural domains among subclasses. The homeobox KN domain, located at the C-terminus, plays a critical role in recognizing the promoter regions of downstream target genes and contributes to the regulation of gene transcription. Upstream of the HD domain, the ELK domain is proposed to act as a nuclear localization signal, while also participating in transcriptional repression and protein–protein interactions. At the N-terminus, the MEINOX domain comprises two subdomains, KNOX1 and KNOX2, which are involved in inter-protein interactions, homodimerization, and the modulation of target gene binding (Sakamoto et al., 1999; Scofield and Murray, 2006). The absence of these two domains is a defining structural feature of the M subclass within the *KNOX* gene family, whereas class I and class II members contain all four conserved structural domains.The presence of the KNOX1 and KNOX2 domains in the LOC101500499 protein sequence, together with the absence of the ELK and HD domains, confirms that the chickpea *homeotic protein knotted-1-like* gene belongs to the M subclass.

## Conclusions

In this study, we adopted an integrative approach to elucidate the genetic basis underlying compound leaf formation in *C. arietinum*. Through the development of a pair of near-isogenic lines (NILs) derived from recombinant lines exhibiting residual heterozygosity for leaf type, combined phenotypic and genotypic analyses identified a *homeotic knotted-1-like* (*KNOX*) gene as a strong candidate involved in the regulation of this trait. Detailed sequence analysis revealed a single-base deletion within the open reading frame, resulting in a frameshift that alters the amino acid sequence and introduces a premature stop codon in genotypes exhibiting the simple-leaf phenotype. Expression analyses supported these findings, and the development of a KASP marker based on this deletion enabled reliable allelic discrimination between compound- and simple-leaf individuals, with a high level of accuracy. The classification and structural features of the predicted protein identify it as a member of the M subclass within the *KNOX* gene family. Future investigations aimed at elucidating the interactions of this gene with additional regulatory genes and signaling pathways will provide valuable insights into the molecular mechanisms governing compound leaf development in chickpea.

## Data Availability

The data presented in this study are publicly available. The data can be found here: https://www.ebi.ac.uk/ena/browser, accession PRJEB102951.
