# Supplementary material for: Identification and characterization of the KNOTTED1-like homeobox as an active regulator of leaf development in chickpea
Source: Front Plant Sci. 2026 Mar 17;17:1803976. doi: 10.3389/fpls.2026.1803976 (PMC13036137; doi:10.3389/fpls.2026.1803976)

Supplementary Figure S2. Multiple sequences alignment from *Cicer arietinum*, *Medicago truncatula* and *Arabidopsis thaliana* was performed using Geneious software. The conserved domain of the KNOX gene family are indicated.

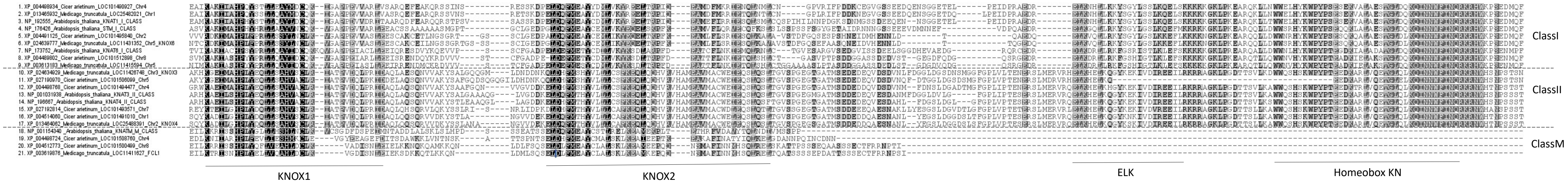

Supplement: Supplementary file 2 [file Image2.pdf]
