# Supplementary material for: Identification and characterization of the KNOTTED1-like homeobox as an active regulator of leaf development in chickpea
Source: Front Plant Sci. 2026 Mar 17;17:1803976. doi: 10.3389/fpls.2026.1803976 (PMC13036137; doi:10.3389/fpls.2026.1803976)

Supplementary Figure S1. Alignment of the mRNA sequence (XM_004512716.4) and the CDS sequence for compound (XP_004512773.1_CL) and simple (XP_004512773.1_SL) leaf. The position of the primer pairs used in this study, the exon and intron, the domain and the translation of the nucleotides are shown. The gap indicates the deletion of a nucleotide found in the simple leaf genotypes.

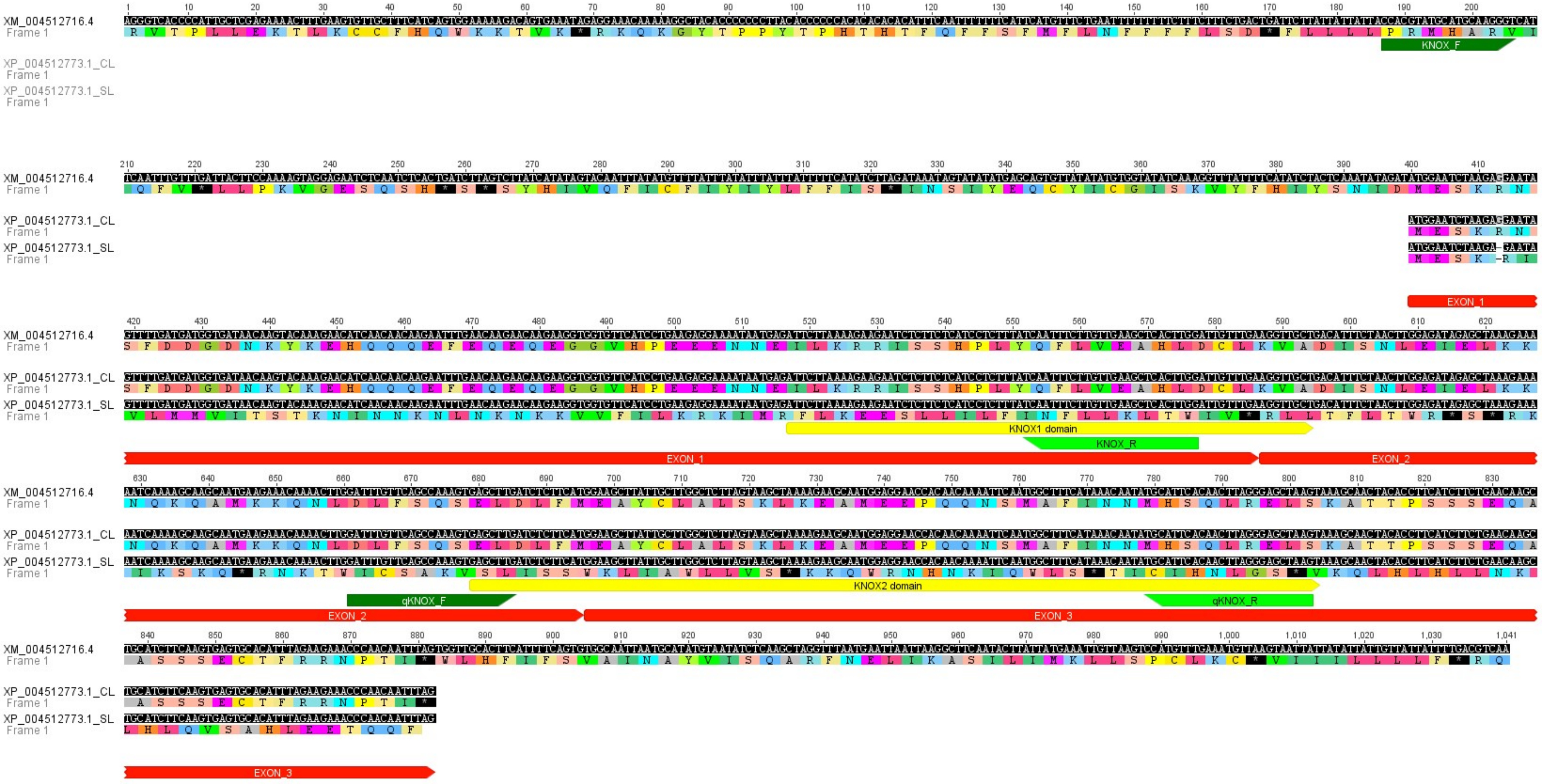

Supplement: Supplementary file 1 [file Image1.pdf]
